# DNA barcoding and morphological analysis for rapid identification of most economically important crop-infesting Sunn pests belonging to *Eurygaster* Laporte, 1833 (Hemiptera, Scutelleridae)

**DOI:** 10.3897/zookeys.706.13888

**Published:** 2017-10-04

**Authors:** Mikhail Y. Syromyatnikov, Victor B. Golub, Anastasia V. Kokina, Victoria A. Soboleva, Vasily N. Popov

**Affiliations:** 1 Voronezh State University, 1 Universitetskaya pl., Voronezh, 394018, Russia

**Keywords:** DNA barcoding, *Eurygaster*, morphological analysis, PCR-RFLP, rapid identification, Sunn pests

## Abstract

The genus *Eurygaster* Laporte, 1833 includes ten species five of which inhabit the European part of Russia. The harmful species of the genus is *E. integriceps*. *Eurygaster* species identification based on the morphological traits is very difficult, while that of the species at the egg or larval stages is extremely difficult or impossible. *Eurygaster integriceps*, *E. maura*, and *E. testudinaria* differ only slightly between each other morphologically, *E. maura* and *E. testudinaria* being almost indiscernible. DNA barcoding based on COI sequences have shown that *E. integriceps* differs significantly from these closely related species, which enables its rapid and accurate identification. Based on COI nucleotide sequences, three species of Sunn pests, *E. maura*, *E. testudinarius*, *E. dilaticollis*, could not be differentiated from each other through DNA barcoding. The difference in the DNA sequences between the COI gene of *E. integriceps* and COI genes of *E. maura* and *E. testudinarius* was more than 4%. In the present study DNA barcoding of two *Eurygaster* species was performed for the first time on *E. integriceps*, the most dangerous pest in the genus, and *E. dilaticollis* that only inhabits natural ecosystems. The PCR-RFLP method was developed in this work for the rapid identification of *E. integriceps*.

## Introduction

The genus *Eurygaster* Laporte, 1833 includes ten species, eight of which have been found in Europe and six in Russia (Göllner-Scheiding 2006). Five *Eurygaster* species inhabit the European part of Russia; four of them are grain crop pests: *E. integriceps* (Puton, 1881), *E. maura* (Linnaeus, 1758), a nominative subspecies of *E. testudinaria* (Geoffroy, 1785), and a nominative subspecies of *E. austriaca* (Schrank, 1776). These species, in particular *E. integriceps* and *E. maura*, reproduce in high numbers on grain crops and considerably reduce crop productivity. Thus, an infestation of Sunn pests (*E. integriceps*, *E. maura*, and *E. testudinaria*) might result in a 20–30% yield loss for barley and a 50–90% yield loss for wheat (Gul et al. 2006). Furthermore, it greatly reduces the baking quality of the flour due to gluten degradation by proteolytic enzymes (Darkoh et al. 2010, Konarev et al. 2013).

**Figure 1.**
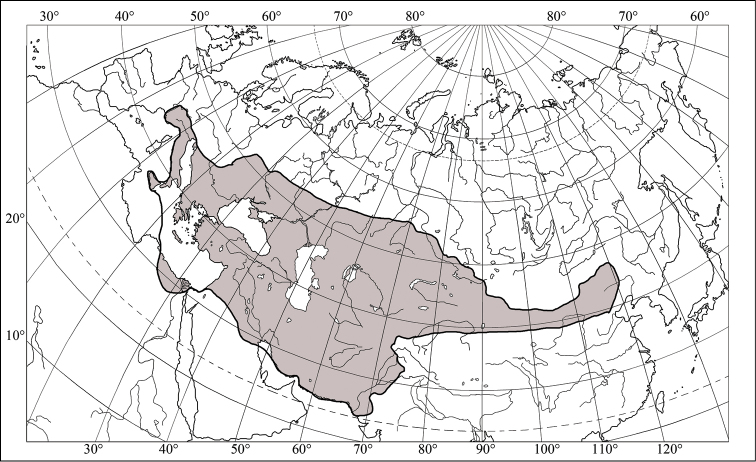
Range of *Eurygaster integriceps* (Puton, 1881) (after Göllner-Scheiding 2006 and Vinokurov et al. 2010).

**Figure 2.**
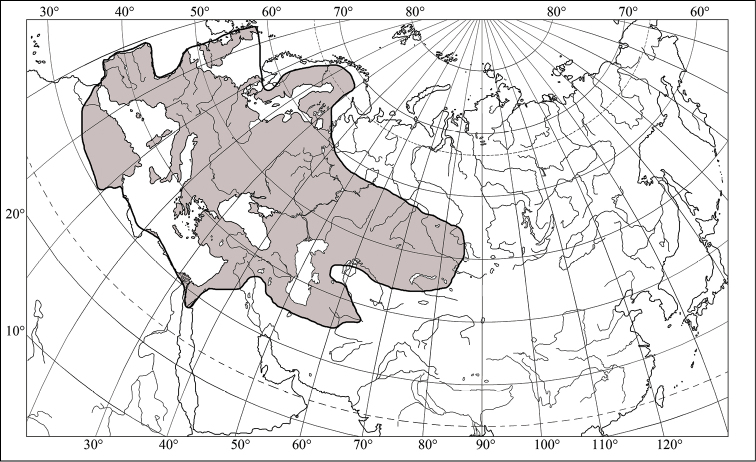
Range of *Eurygaster maura* (Linnaeus, 1758) (after Göllner-Scheiding 2006 and Vinokurov et al. 2010).

**Figure 3.**
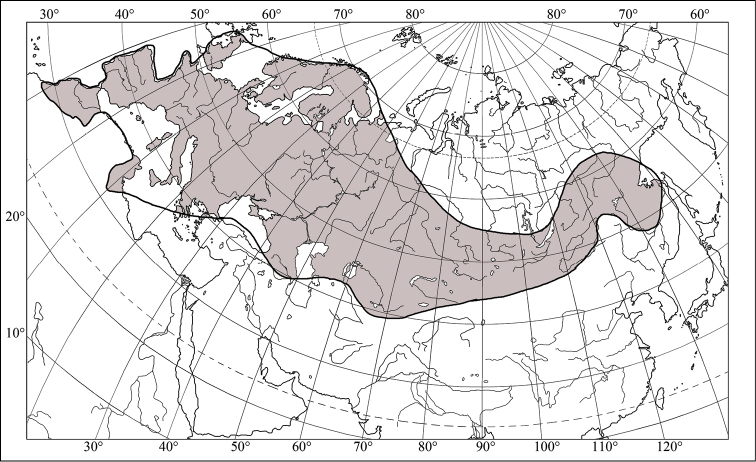
Range of *Eurygaster testudinaria* (Geoffray, 1758) (after Göllner-Scheiding 2006 and Vinokurov et al. 2010).

**Figure 4.**
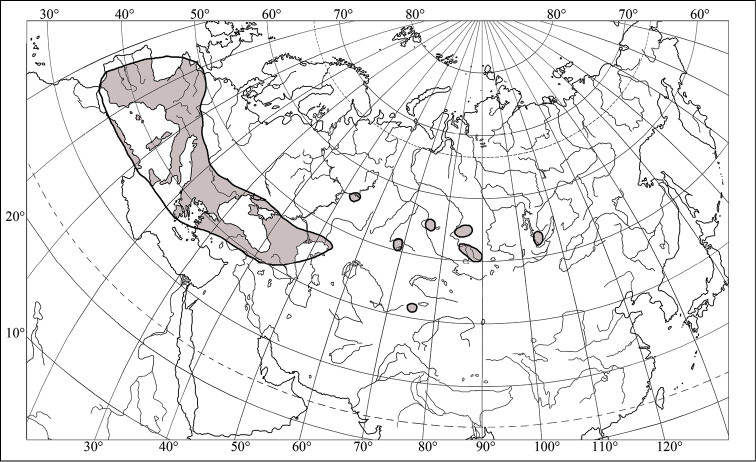
Range of *Eurygaster dilaticollis* Dohrn, 1860 (after Göllner-Scheiding 2006 and Vinokurov et al. 2010).

*Eurygaster integriceps* is the most damaging bread wheat and durum wheat pest in western and central Asia and Eastern Europe (Radjabi 1994, Gul et al. 2006). It is widespread in south-eastern Europe, central Asia, and the Middle East (Fig. 1). The range of *E. maura* covers central and southern Europe (including European Russia), Caucasus, Turkey, North Africa (Algeria, Morocco, Tunisia), and central Asia (Fig. 2). *Eurygaster testudinaria* is a transpalaearctic species (Fig. 3). *Eurygaster dilaticollis* is distributed in central and southern Europe (including the middle and southern territories of the European part of Russia), Turkey, central Asia, western and eastern Siberia (Göllner-Scheiding 2006) (Fig. 4). *Eurygaster dilaticollis* Dohrn, 1860 inhabits pastures and natural steppe ecosystems and feeds on grass sap. The extent of crop damage by this species has not been evaluated yet. The range of *E. austriaca* covers central and southern Europe, Caucasus, Turkey, North Africa, and central Asia (Kazakhstan). This species is rare in Eastern Europe.

The species representation and the numbers of Sunn pests constantly changes following changes in climatic conditions, structure of sown areas, and crop cultivation technologies (Critchley 1998). Global climatic changes in the future can expand the habitat of the most dangerous species, *Eurygaster integriceps* (Aljaryian et al. 2015). This creates a need for a rapid and accurate identification of *Eurygaster* species (particularly *Eurygaster integriceps*) infesting crops for the early detection of the pest in a new territories and the use of preventive measures. Until now, such identification has been based mostly on analyses of external morphological features, including male and female genitalia. This requires long-term making of microscopic preparations and study of many specimens in the samples. Moreover, specimens collected from the same area almost always contain representatives of 2–3 *Eurygaster* species, and the insignificant external morphological differences between *E. integriceps*, *E. maura*, *E. dilaticollis*, and *E. testudinaria* prevent their accurate identification (unpublished data).

Recently, molecular genetic methods, in particular DNA barcoding and phylogenetic analysis, have become very popular for revealing the taxonomic affiliation of organisms. DNA barcoding has proven itself as a valuable tool for identifying organisms (Hebert et al. 2003a, Ferri et al. 2009). It includes the amplification and sequencing of a gene fragment and its comparison with the corresponding sequences in existing databases, such as Boldsystems (http://www.boldsystems.org)and GenBank (https://www.ncbi.nlm.nih.gov/genbank). The gene commonly used for barcoding is mitochondrial cytochrome c oxidase subunit I (COI) for animals (Hebert et al. 2003b). DNA barcoding might allow rapid identification of crop pests, which will provide the basis for differential treatment of crops. It should be noted that DNA barcoding of *E. maura* and *E. testudinaria* was carried out earlier (Park 2011).

A significant advantage of molecular methods is the possibility of identifying pests at different stages (egg or larval), i.e., when morphological identification is extremely difficult or impossible. Molecular identification might be useful for the early detection of pests on cereal crops, since the larvae of *E. integriceps* during stages I–III are difficult or impossible to distinguish from other species of the same genus.

Morphological features of *Eurygaster* species were investigated in this study. The variations in the nucleotide sequence of the COI gene of *Eurygaster* species were identified. DNA barcoding of two *Eurygaster* species has been performed for the first time on the most dangerous grain crop Sunn pests *E. integriceps* and *E. dilaticollis*, which inhabits natural steppe ecosystems. We have developed a method for the rapid identification (PCR-RFLP) of the pest *E. integriceps* based on COI sequences.

## Materials and methods

### Insect resources

Specimens for morphological and molecular genetic studies were collected by the authors in 2013–2015 in three regions of Russia. Specimens of *E. integriceps*, *E. maura*, and *E. testudinaria* were collected from the environments of Voronezh city (N51°40', E39°12'; altitude, 150–160 m); Specimens of *E. dilaticollis* were collected in the Teberda State Nature Reserve, north-west Caucasus (43°27'N, 41°45'E; alt., 1350–1600 m) and in the southern Ural State Reserve, southern Urals, (54°11'N, 57°37'E; alt., 285–300 m). Because of the absence of *E. austriaca* in our collections from cereal crops and natural ecosystems at these points in the European part of Russia during the study period, and the absence of this species as a cereal pest in the vast territory of the European part of Russia, DNA barcoding of this species has not been made by us. The collected specimens from the four species of *Eurygaster* species were stored at the Voronezh State University. Insects were collected in areas containing cereals and wild grasses with an insect collecting net. The bugs that were caught were placed individually in test tubes with 96% ethanol, labeled, and transported on the same day to the laboratory. Prior to analyses the samples were stored at - 20 °C to slow the degradation of DNA. The morphological features of *Eurygaster* species were studied using a collection of more than 800 *Eurygaster* specimens from different regions of Eurasia stored at the Zoological Institute of the Russian Academy of Sciences (St. Petersburg).

### Morphological analysis

Specimen preparation and morphological studies were performed using an MBS-10 binocular light microscope. Photographs of the specimens were taken with a Leica DFC495 camera mounted on a Leica M205C binocular microscope. Image processing and analyses were performed using the Leica Application Suite v4.5 software. Drawings of genitalia of male *Eurygaster* species were made using a RA-6 drawing apparatus after genitalia isolation and treatment with 4% KOH (Golub et al. 2012). Morphological identification was carried out according to the previously developed identification keys (Kiritshenko et al. 1951b, Stichel 1959-1962, Kerzhner and Jaczewski 1964, Golub 1980).

### DNA extraction and barcoding

DNA was isolated from the legs of the specimens with a ZR Tissue & Insect DNA MicroPrep kit (Zymo Research, USA). Voucher specimens are stored in the department of Ecology and Systematics of Invertebrates of Voronezh State University. Polymerase chain reaction was performed with an Eppendorf MasterCycler Personal cycler. Each PCR reaction mixture contained 2.5 µl of 10x reaction buffer (Evrogen, Russia), 1 µl of 10 mM dNTPs, 1 µl of 10 µM forward primer, 1 µl of 10 µM reverse primer, 3 µl of 25 mM Mg2^+^, 1 µg of template DNA, 2.5 units of thermostable *Taq* DNA polymerase (Evrogen, Russia), and deionized water (up to 25 μl). The PCR regime included initial denaturation at 94 °C for 3 min; 35 cycles of denaturation at 94 °C for 30 s, annealing at 51 °C for 30 s, elongation at 72 °C for 45 s; and final elongation at 72°C for 10 min. The primers used were: forward LepF1 5'-ATTCAACCAATCATAAAGATATTGG (Hebert 2004, Wilson 2012), reverse LepR1 5'-TAAACTTCTGGATGTCCAAAAAATCA (Hebert 2004, Wilson 2012). Also, we used EurG-f 5’-GAATATGAGCCGGAATAGTAGGA and EurG-r 5’-ATGTGTTGAAGTTACGGTCA primers, developed by us. PCR products were separated by electrophoresis in 2% agarose gel, stained with ethidium bromide, and visualized with a TCP-20LM transilluminator at 312 nm. The size of the PCR products was determined using 100+ DNA length standards (Evrogen, Russia).

PCR products were purified from the agarose gel with a commercially available Cleanup Standard kit (Evrogen, Russia) and sequenced with an Applied Biosystems 3500 genetic analyzer using the BigDye Terminator v3.1 Cycle Sequencing Kit. DNA barcoding primers (LepF1, LepR1, EurG-r and EurG-f) were used for sequencing. Sequence alignment was performed with the Clustal Omega tool (http://www.ebi.ac.uk/Tools/msa/clustalo/). Sequences were translated into amino acid sequences to verify that it was free of stop codons and gaps with EMBOSS Transeq (http://www.ebi.ac.uk/Tools/st/emboss_transeq/). Phylogenetic analysis was carried out using Mega 6 (Center for Evolutionary Medicine and Informatics, USA) software. The sequences were truncated to 479 bp. Pairwise genetic distances between specimens were calculated using the Kimura 2 Parameter (K2P) model (Kimura 1980). The K2P model provides a substitution framework with free parameters for both transitions and transversions, accounting for the likely higher substitution rate of transitions in mitochondrial DNA. The gene tree reconstruction was inferred using the Neighbor-Joining method (Saitou and Nei 1987). The percentage of replicate trees in which the associated taxa clustered together in the bootstrap test (500 replicates with pairwise deletion of gaps/missing data and inclusion of all substitutions (transitions and transversions)) are shown next to the branches (Felsenstein 1985). The tree is drawn to scale, with branch lengths in the same units as those of the evolutionary distances used to infer the phylogenetic tree. The evolutionary distances were computed using the Kimura 2-parameter method and are in the units of the number of base substitutions per site. The analysis involved 35 nucleotide sequences. All positions with less than 95% site coverage were eliminated. That is, fewer than 5% of alignment gaps, missing data, and ambiguous bases were allowed at any position. There were a total of 479 positions in the final dataset. Gene tree reconstruction was conducted in MEGA6 (Tamura et al. 2013). *Odontotarsus purpureolineatus* (Rossi, 1790) (Hemiptera: Scutelleridae) was chosen as outgroup. Estimates of evolutionary divergence between groups were conducted using the Kimura 2-parameter model (Saitou and Nei 1987).

### Design of primers and probes

Primer and probe design for the fast identification of *Eurygaster* species was performed according to the most appropriate of the following factors: 1. primer length between 18 bp and 30 bp; 2. no distinct hairpin structure and dimers; 3. GC% from 20% to 80% for primers and probes; 4. the minimum G/C content at the 3 ‘end of the primers; 5. minimum identical nucleotides together in probes; 6. the 5’-end of probes must not be G; 7. PCR-product size: from 50 bp to 200 bp; 8. the annealing temperature of the probes must be at least 5 °C above the annealing temperature of the primers; 9. several SNPs (for *Eurygaster integriceps* and other species of the same genus) at the DNA-probe hybridization site.

### PCR-RFLP

Analysis of suitable restriction enzymes for species differentiation was performed using theoretical diagrams of DNA digestion by enzymes, available from http://www.sibenzyme.com/products/restrictases. The PCR product was obtained with the forward (EurG-f 5’-GAATATGAGCCGGAATAGTAGGG) and reverse (EurG-r 5’-ATGTGTTGAAGTTACGGTCA) primers that were designed according to the sequencing data. PCR products (10 µl) were digested in the reaction mixture containing 1.5 µl of 10X reaction buffer and 10 U of restriction endonuclease Bst2UI, AhlI and PsiI (Sibenzym, Russia) in a total volume of 15 µl. The mixture was incubated for 2 h at 37 °C, and the enzyme was then inactivated at 75 °C for 15 min. The digestion products were visualized by electrophoresis with bromide ethidium in 2% agarose gel.

### Ethics statement

The collection of *Eurygaster* pest species from the territory of Teberda State Nature Reserve (north-west Caucasus) was carried out under the agreement regarding the collaboration of scientific research between Voronezh State University and Teberda State Nature Reserve. The collection of *Eurygaster* pest species from the territory of Southern Ural State Reserve (southern Urals) was carried out under the agreement regarding the scientific research collaboration between Voronezh State University and Southern Ural State Reserve. These agreements include the procedures for harvesting, collection, analysis, and publishing of the obtained results for different taxonomic groups of insects, including the pests. The collection of *Eurygaster* pest species from the suburbs of Voronezh city was carried out at the “Venevitinovo”, biological station, which is a structural part of Voronezh State University, in accordance with internal university bioethical rules.

## Results

### Specimens

184 samples of various species of bugs were collected during this study. Morphological and molecular analysis (DNA barcoding and PCR-RFLP) were performed with adult specimens that were not damaged during collection (Table 1).

**Table 1.** Collected and analyzed specimens.

N	Species	Locality, coordinates	Data of collection	Primers for DNA barcoding	Voucher number	GenBank reference, product length
1.1	*E. integriceps*	Voronezh city, 51°40'N, 39°12'E altitude, 150–160 m	June 2015	LepF1/LepR1	VSU_003	KR105371.1 658 bp
1.2				LepF1/LepR1	VSU_010	KU760764.1 658 bp
1.3				EurG-f/LepR1	VSU_Int_1	KX708594.1 576 bp
1.4				EurG-f/LepR1	VSU_Int_2	KX708595.1 576 bp
1.5				EurG-f/LepR1	VSU_Int_3	KX708596.1 576 bp
1.6			June 2016	EurG-f/LepR1	VSU_Int_4	KX708597.1 576 bp
1.7				EurG-f/LepR1	VSU_Int_5	KX708598.1 576 bp
1.8				EurG-f/LepR1	VSU_Int_6	KX708599.1 576 bp
1.9				EurG-f/EurG-r	VSU_Int_7	KX708600.1 505 bp
1.10–1.20		Voronezh region	June 2016	None	VSU_Int_8 to VSU_Int_18	Verified morphological and PCR-RFLP
2.1	*E. maura*	Voronezh city, 51°40'N, 39°12'E altitude, 150–160 m	June 2015	LepF1/LepR1	VSU_008	KU760762.1 658 bp
2.2		Voronezh region	June 2016	EurG-f/LepR1	VSU_Mau_2	KX708603.1 588 bp
2.3–2.12			June 2016	None	VSU_ Mau_3 to VSU_ Mau _12	Verified morphological and PCR-RFLP
3.1	*E. testudinaria*	Voronezh city, 51°40'N, 39°12'E altitude, 150–160 m	June 2015		VSU_007	KU760761.1 658 bp
3.2			June 2016	EurG-f/EurG-r	VSU_Tes_1	KX708605.1 504 bp
3.3				EurG-f/EurG-r	VSU_Tes_2	KX708606.1 505 bp
3.4				EurG-f/LepR1	VSU_Tes_3	KX708607.1 573 bp
3.5				EurG-f/LepR1	VSU_Tes_4	KX708608.1 588 bp
3.6–3.15		Voronezh region	June 2016	None	VSU_Tes_5 to VSU_Tes_14	Verified morphological and PCR-RFLP
4.1	*E. dilaticollis*	North-West 105 Caucasus, 43°27'N, 41°45'E; alt., 1350–1600 m	June 2015	EurG-f/LepR1	VSU_009	KU760763.1 613 bp
4.2		Southern Ural, 54°11'N 57°37’; E alt., 285–300 m	June 2014	EurG-f/EurG-r	VSU_Dil_1	KX708601.1 502 bp
4.3–4.12				None	VSU_Dil_2 to VSU_Dil_11	Verified morphological and PCR-RFLP

### Morphological studies

**Figure 5.**
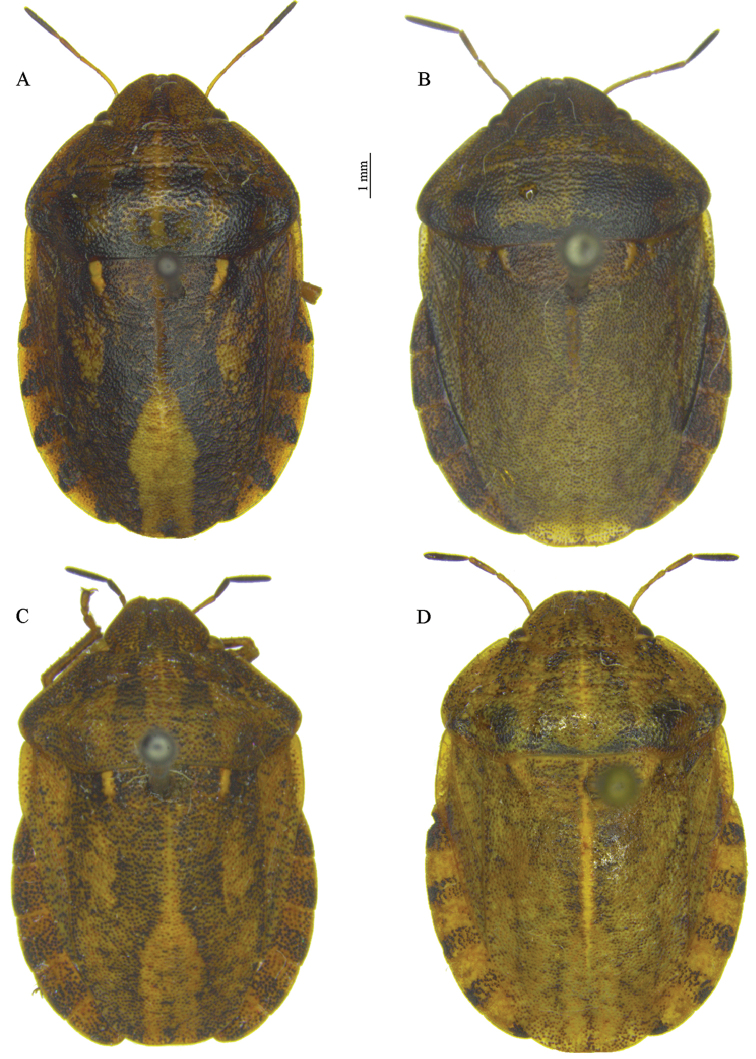
Species of the genus *Eurygaster* Laporte, general view: **A** *E. integriceps* (Puton) **B** *E. maura* L. **C** *E. testudinaria* (Geoffroy) **D** *E. dilaticollis* Dohrn. Specimens **A–C** were collected in the Voronezh Region; specimen D was from the Teberda Nature Reserve, Caucasus.

**Figure 6.**
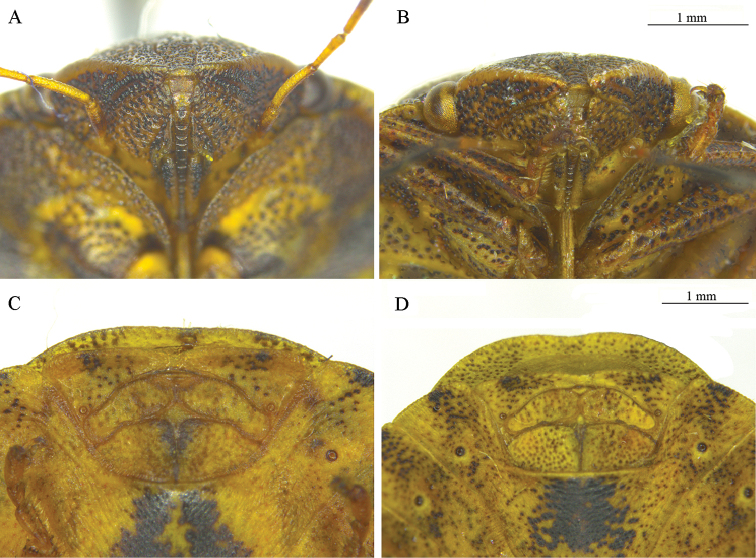
Head, anterior view (**A, B**) and female median genital plates (**C, D**) of *E. maura* L. (**A, C**) and *E. testudinaria* (Geoffroy) (**B, D**).

**Figure 7.**
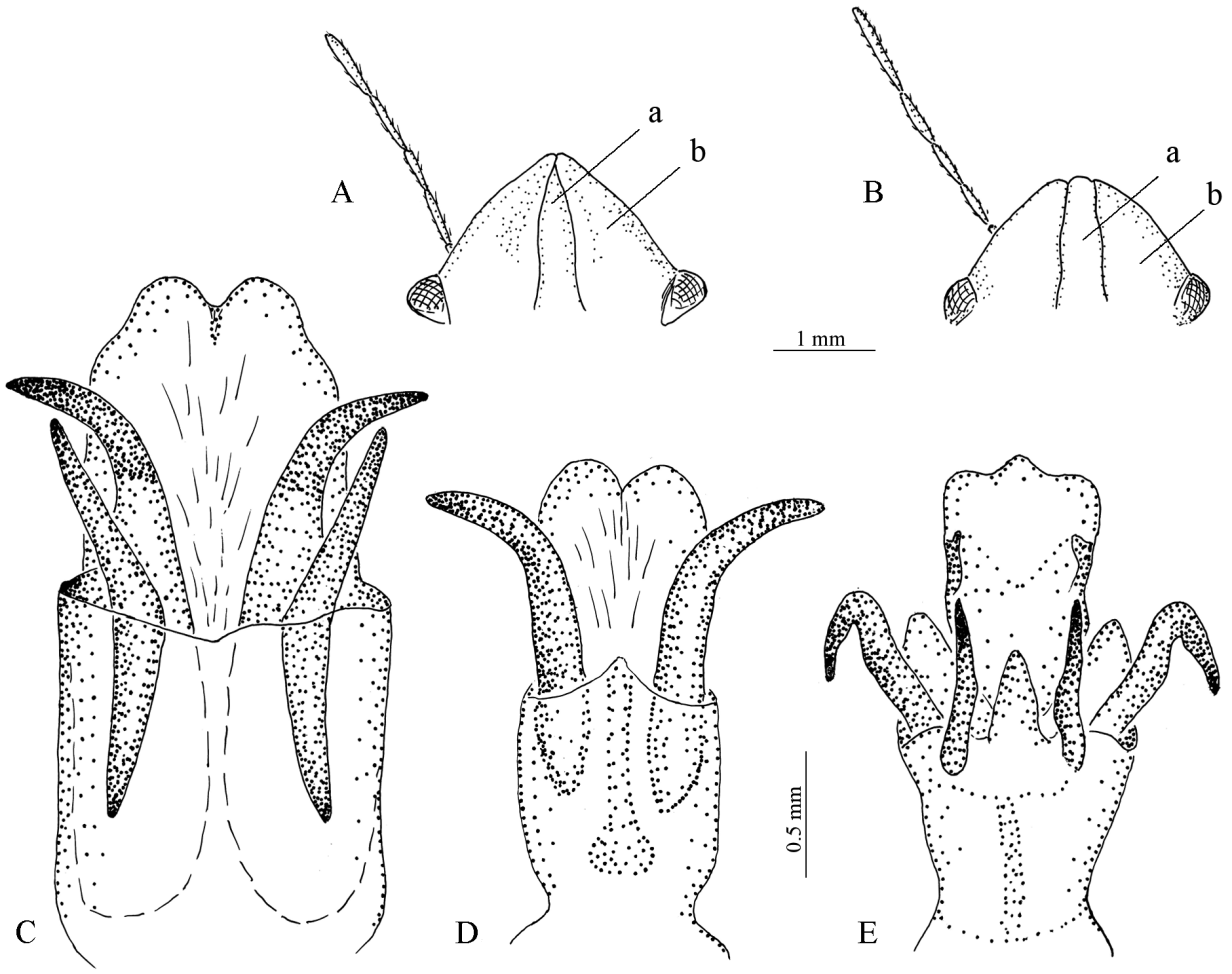
Structural details of *Eurygaster* Laporte species (a, clypeus; b, jugal plate): **A** *E. austriaca* (Schrank), head, dorsal view **B** *E. integriceps* (Puton), dorsal view **C** *E. integriceps*, aedeagus **D** *E. maura* L., aedeagus **E** *E. testudinaria* (Geoffroy), aedeagus (after Golub, 1980, with changes).

The morphological features of the *Eurygaster* species proposed earlier by different authors, including the co-author of the present work were used (Batzakis 1972, Golub 1980, Kerzhner and Jaczewski 1964, Vinogradova 1959), with the addition of the main morphometric features of the three most dangerous cereals pests in eastern European Russia, *E. integriceps*, *E. maura*, and *E. testudinaria* (Table 3). The main morphological differences between these species are shown in Table 2 and Figs 5–7.

**Table 2.** Morphological features of *E. integriceps*, *E. maura, E. dilaticollis*, and *E. testudinaria*.

Species	Morphological features					
	Pronotum lateral margins/lateral angles	Presence of medial keel on scutellum/ tubercles near scutellum anterior angles	Apices of jugal plates	Female median genital plates	Number of sclerotized hooks of aedeagus	Body length, mm
*E. integriceps* (Fig. 5A)	Slightly convex/rounded, not salient laterally of the base of hemelytra	Yes/yes	In the plane of clypeus apex or insignificantly above it	Almost reaching lateral margins of abdominal segment VII	4	9.8–13.0
*E. maura* (Fig. 5B)	Straight or slightly concave/rounded, not or barely noticeable salient laterally of the base of hemelytra	No/no	In the plane of clypeus apex or insignificantly above it	Reaching or almost reaching lateral margins of abdominal segment VII	2	8.0–11.5
*E. testudinaria* (Fig. 5C)	Straight or slightly concave/acuminate, slightly salient laterally of the base of hemelytra	No or barely expressed/no	Distinctly or insignificantly above the plane of clypeus apex	Distinctly not reaching or almost reaching lateral margins of abdominal segment VII	4	8.0–10.5
*E. dilaticollis* (Fig. 5D)	Slightly convex, rounded/ barely noticeable salient	Yes/no	In the plane of clypeus apex	Not reaching lateral margins of abdominal segment VII	6	8.0–10.5

*Eurygaster austriaca* significantly differs from the above-mentioned three species: the frontal part of its head clypeus is covered by jugal plates (Fig. 7A). *Eurygaster dilaticollis* differs from other species by a short pronotum that is not much than the head (Fig. 5D).

Morphometric parameters on the base of measurements of both sexes in the samples of three cereals pests from the Voronezh Region are given in Table 3.

Morphometric parameters on the base of measurements of specimens of both sexes in the samples of three cereals pests from the Voronezh Region are given in Table 3.

**Table 3.** Morphometric data of *E. integriceps*, *E. maura*, and *E. testudinaria* from Voronezh region.

Species		Body length; limits; average, mm	Body width; limits; average, mm	Pronotum length; limits; average, mm	Pronotum width, limits; average, mm	Body length / body width; limits; average	Pronotum width / pronotum length; limits; average
*Eurygaster integriceps*	♂♂	9.80–12.00; 10.76±0.132	6.80–7.30; 7.04±0.030	3.00–3.30; 3.13±0.018	6.70–6.90; 6.72±0.018	1.42–1.69; 1.53±0.019	2.00–2.33; 2.17±0.020
	♀♀	11.90–13.00; 12.30±0.090	6.90–7.60; 7.24±0.042	3.30–3.60 3.46±0.018	6.60–7.00; 6.82±0.024	1.62–1.80; 1.70±0.014	1.91–2.06; 1.97±0.009
*Eurygaster maura*	♂♂	8.30–10.20; 8.93±0.132	5.90–6.50; 6.21±0.036	2.40–3.00; 2.64±0.036	5.50–5.80; 5.66±0,018	1.31–1.57; 1.44±0.016	1.09–2.28 2.14±0.026
	♀♀	8.90–11.50; 10.00±0,156	6.30–6.70; 6.47±0,030	2.70–3.10; 2.96±0.024	5.70–6.70; 5.87±0.060	1.43–1.74; 1.55±0.018	1.87–2.31; 1.98±0.026
*Eurygaster testudinaria*	♂♂	8.70–9.80; 9.24±0.066	5.60–6.00; 5.92±0.036	2.70–3.10; 2.86±0.024	5.30–5.60; 5.40±0.018	1.47–1.64; 1.56±0.010	1.80–1.96; 1.89±0.010
	♀♀	9.50–10.50; 9.99±0.060	6.00–6.70; 6.47±0.042	2.60–3.10; 2.85±0,030	5.70–6.30 6.02±0.042	1.49–1.58; 1.54±0.006	2.00–2.18; 2.1±0.011

### DNA barcoding

DNA isolated from collected Sunn pest specimens was used for COI gene amplification. It was found that the universal primers LepF, LepF2_t1 and MHemF, commonly used for the identification of insects (Wilson 2012), had a very low specificity toward the isolated DNA of these insects.

658 bp length DNA sequences (Folmer region) obtained with LepF1/LepR1 primers were registered in the GenBank database under the numbers presented in Table 1. The sequences are also registered in the Bold System database with the following Barcode Index Numbers (BINs) assigned: *E. integriceps* – BOLD:AAZ6788; *E. maura* – BOLD:AAZ3231; *E. testudinaria* – BOLD:AAZ3231; *E. dilaticollis* – BOLD:AAZ3231.

Analysis of the nucleotide sequences of COI genes from the three main pests of crops in Eastern Europe, *E. integriceps*, *E. maura*, and *E. testudinaria*, has shown that the difference between the COI gene of *E. integriceps* and that of the two other species was more than 4%.

We failed to amplify the COI gene from *E. dilaticollis* when using either LepF1/LepR1 primer pair or any of the other primer pairs commonly used for COI amplification (LCO/HCO, LCO_t1/HCO_t1, MLepF1/MLepR1, as well as combinations of these primers). The only two primer pairs that successfully produced the required PCR product were EurG-f /EurG-r and EurG-f /LepR1; however, the amplicon length in this case was shorter than 613 bp. Its nucleotide sequence was the same as those from *E. maura* and *E. testudinaria*. DNA barcoding of *E. dilaticollis* was performed for the first time.

**Figure 8.**
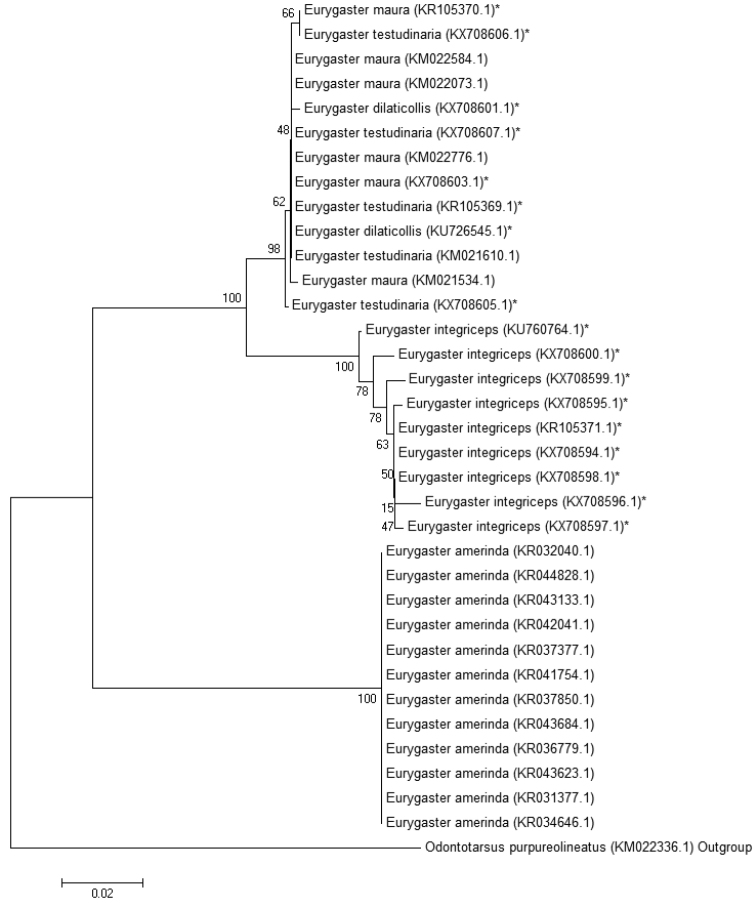
Neighbor joining analysis of COI gene sequences from *Eurygaster* species. * - sequences obtained in this work.

A Neighbor-joining (NJ) tree was shown to be a useful clustering method for large datasets (Yang and Rannala 2012, Tamura et al. 2004). We have reconstructed a phylogenetic tree that reflects genetic distances between *Eurygaster* species using Kimura 2-parameter algorithm and the COI gene sequences of *Eurygaster* species obtained by us as well as all *Eurygaster* species sequences available in the GenBank database (Fig. 8).

The genetic distance between the *E. integriceps* species and the group species that includes the 3 species (*E. maura*, *E. testudinaria* and *E. dilaticollis*) was 0.049. The genetic distance between the *E. integriceps* species and *E. austriaca* was 0.121. The within-group mean distance for *E. integriceps* was 0.007, for *E. maura* 0.001, and for *E. testudinaria* it was 0.002.

### Development of a PCR method for the rapid identification of *E. integriceps*

Considering the fact that the COI nucleotide sequence of *E. integriceps* differs significantly from those of *E. maura* and *E. testudinaria*, a method for its rapid identification has been developed using an analysis of the nucleotide regions of cytochrome oxidase (COI) and two identification methods have been tested: PCR with TaqMan probes and PCR-RFLP (Restriction Fragment Length Polymorphism). Conservative DNA sequences within each species were identified. First, two sets of PCR primers and probes were developed by identifying the SNP-carrying fragments within the COI gene sequence as sites for probe and primer annealing (Table 4).

**Table 4.** Primer/probe set for species identification.

Species	Primer/probe set	
*E. maura* *E. testudinaria* *E. dilaticollis*	Set 1	forward primer: MTI-f 5’-AGCAGGTGTTTCCTCAATCTTAG Probe: FAM-ACCCATTGGTATAACACCTGAACGAACCCCA-BHQ1 Reverse primer: MT-r 5’-AGTAATAATGCGGTAATTCCAACTG Product length – 129 bp
*E. integriceps*		forward primer: MTI-f 5’-AGCAGGTGTTTCCTCAATCTTAG Probe: FAM-CGACCCGTTGGTATAACACCTGAACGGATCC-BHQ1 Reverse primer: I-r – 5’-AGTAATAATGCAGTAATTCCAACTG Product length – 129 bp
*E. mauraE. testudinaria* *E. dilaticollis*	Set 2	MT1-f: 5’-ATCAGTTGGAATTACCGCATTATTA Probe: FAM-TACTACTATCATTGCCAGTACTAGCCGGAGC-BHQ1 Reverse primer: MTI1-r – 5’-ATGTGTTGAAGTTACGGTCA Product length – 95 bp
*E. integriceps*		I1-f: 5’-ATCAGTTGGAATTACTGCATTATTA Probe: FAM-TGCTACTATCACTACCAGTACTAGCAGGAGC-BHQ1 Reverse primer: MTI1-r: 5’-ATGTGTTGAAGTTACGGTCA Product length – 95 bp

Despite optimization of PCR conditions (temperature, DNA template concentration, primer/probe concentrations), we failed to achieve 100% species-specific identification for either *E. integriceps* or *E. maura*/*E. testudinaria*. Overall, out of nine PCR reactions, nonspecific primer and probe annealing (i.e. annealing of primers and probe specific for one of *Eurygaster* species on DNA of other species) was observed in two reactions.

Another method for the express identification of *E. integriceps* is PCR-RFLP. Preliminarily, COI nucleotide sequences were analyzed from various *Eurygaster* species for the presence of restriction enzyme sites that would be different in these species and produce cleavage products suitable for electrophoretic analysis in agarose gel. The possibility of using more than 100 restriction enzymes was examined and three restriction enzymes were chosen. The reaction products for these enzymes are well separated in agarose gel and have specific patterns for the *E. maura*/*E. testudinaria*/ *E. dilaticollis* and *E. integriceps* considering intraspecific variability. The selected restriction enzymes are shown in Table 5.

**Table 5.** Restriction enzymes for PCR-RFLP and expected lengths of the 585 bp COI fragment cleavage products.

Restriction enzyme	Recognition site	Fragments for *E. integriceps*, bp	Fragments for *E. maura*/*E. testudinaria*/ *E. dilaticollis*, bp
Bst2UI	CCWGG	364, 221	585
PsiI	TTATAA	435, 150	435, 91, 59
AhlI	ACTAGT	317, 268	317, 175, 93

**Figure 9.**
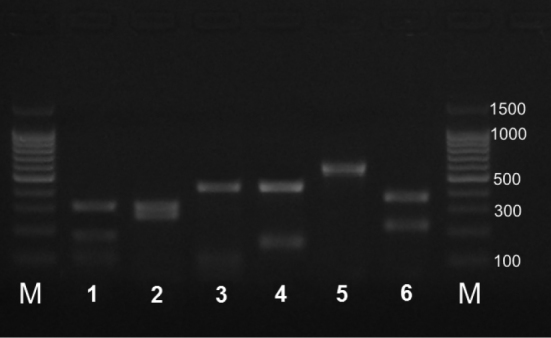
Restriction fragments of COI PCR products (restriction enzyme, species): **1** *AhlI*, *E. maura* or *E. testudinaria* **2** *AhlI*, *E. integriceps* **3** *PsiI*, *E. maura* or *E. testudinaria* **4** *PsiI*, *E. integriceps* **5** *Bst2UI*, *E. maura* or *E. testudinaria* **6** *Bst2UI*, *E. integriceps*; M, 100 bp DNA ladder.

To obtain a PCR fragment for restriction analysis forward (EurG-f 5’-GAATATGAGCCGGAATAGTAGGG) and reverse (EurG-r 5’-ATGTGTTGAAGTTACGGTCA) primers were used that yielded a 585-bp PCR product. The primers LepF1/LepR1 could not be used in this case because of the low specificity of the LepF1 primer for *Eurygaster* species. Cleavage of the obtained PCR product resulted in DNA fragments of predicted sizes for all tested species (Fig. 9).

Eight specimens from each *Eurygaster* species were analyzed by this method and any of the restriction enzymes could be successfully used for identification of *E. integriceps*.

## Discussion

The differences in the sequences of COI gene from *E. integriceps* and other closely related species largely correlate with the morphological differences between these species (Table 1). The body of *E. integriceps* is, on average, larger with slightly rounded lateral edges of the pronotum (Fig. 5). The observed higher intraspecific variation of the COI nucleotide sequence in *E. integriceps* is possibly associated with its significant migratory activity during the periods of preparation for the winter diapause and the exit from it. Such migrations can occur over large distances (up to dozens of kilometers) and can result in mating between organisms from different populations after wintering (Critchley 1998). This might contribute considerably to the exchange of genes between populations.

The similarity between COI nucleotide sequences of *E. maura* and *E. testudinaria* correlates with the high levels of morphological similarity between these species (Table 1). The high variability of external features (especially morphological characteristics of the head, which can often be present in both species) does not allow for the definite identification of specimens from either species. *Eurygaster maura* and *E. testudinaria* can be distinguished based on the number of sclerotized hooks inside the aedeagus. This difference in the fine structure of male genitalia is a result of evolutionary processes aimed at preventing interspecific hybridization. However, in practical terms, species identification based on the internal structure on the aedeagus is difficult at best, if populations are mixed, it is the only way to identify the species.It should be noted that the variability of external morphological characteristics within each of the three main harmful species is high enough to separate them (Table 3). Therefore, for accurate determination of species it is necessary to examine the external features of a series of specimens as well as the characteristics of the genitalia. Accurate morphological identification of the adults of *Eurygaster* species is possible; however, it requires a large number of *Eurygaster* specimens without admixture of another species.

It appears that resolution of the classic DNA barcoding is not sufficient for distinguishing some species with small differences between the two species such as structure of genitalia. Indeed, it is known that DNA barcoding is not always capable of differentiating between closely related species (Whitworth 2007, Will and Rubinoff 2004, Meyer and Paulay 2005). Although it is important to search for other molecular genetic markers for definite identification of *E. maura* and *E. testudinaria*, differentiation between these two species is currently not relevant, since the deleterious effect of both species in southern and Eastern Europe and Asia is much lower compared to that of *E. integriceps*.

The obtained tree has two clearly distant branches. The first one includes five Palaearctic species, *E. integriceps*, *E. maura*, *E. testudinaria*, *E. dilaticollis*. The second branch includes one Nearctic species, *E. amerinda* Bliven, 1956. The genetic distance between these two groups clearly reflects continental disjunction and autochthonous morphogenetic processes that took place within the same genus on two different continents during the Cenozoic. Within the Palaearctic group, a subgroup including *E. maura*, *E. testudinaria*, and *E. dilaticollis* are genetically similar to each other. *Eurygaster maura* and *E. testudinaria* are not always distinguishable. *Eurygaster integriceps* belongs to a separate phylogenetic branch that is closer to the first three species than *E. austriaca* (data not present on tree). The latter is the most distant species, both genetically and morphologically, from the analyzed Palearctic species (Table 1, Figs 5–7). High intraspecific variability was shown for *E. integriceps*. This is consistent with the previous data on the high intraspecific variability postulated in some species of the order Hemiptera (Raupach et al. 2014).

Under the conditions in Eastern Europe and especially the vast territory of southern Russia, Ukraine, central Asia, *E. integriceps* is the most xerophilous and thermophilic species of *Eurygaster* (Critchley 1998). During the emergence of larvae in the early growing season, populations may be represented by several species of this genus and are not easily differentiated. However, the prevalence of *E. integriceps* species is likely to increase much more rapidly than that of other species. In this regard, in order to predict the size of the main *E. integriceps* pest population and prepare the proper treatment with pesticides (earlier treatment with pesticides is needed when *E. integriceps* is identified), monitoring their development and proliferation is necessary. Analyzing the proliferation and the activity of other pest species of the genus *Eurygaster* would not be so important, due to their much lower abundance and less damaging habits. The advantages of the developed PCR-RFLP method for the express identification of *E. integriceps* are its reproducibility, simplicity, and low cost of analysis. It should be noted that this is only a preliminary result and requires tests in populations of Sunn pests from other areas.

The early detection of *E. integriceps* in crops as their primary pest is important in connection with the potential expansion of its habitat, due to global climate change (Aljaryian et al. 2015). Rapid detection of this pest in the new territories will prevent additional loss of yield and, to a certain extent, slow down its invasion and expansion into other areas. A platform for the identification of the pest *Eurygaster integriceps* based on PCR-RFLP that was developed in this study will allow the express detection of the presence of the pest in new areas and avoid false positives results.
